# Nanogels with covalently bound and releasable trehalose for autophagy stimulation in atherosclerosis

**DOI:** 10.1186/s12951-023-02248-9

**Published:** 2023-12-08

**Authors:** Yuan Zhong, Ali Maruf, Kai Qu, Małgorzata Milewska, Ilona Wandzik, Nianlian Mou, Yu Cao, Wei Wu

**Affiliations:** 1 Key Laboratory for Biorheological Science and Technology of Ministry of Education, State and Local Joint Engineering Laboratory for Vascular Implants, Bioengineering College, Faculty of Medicine, Chongqing University, Chongqing, 400030 China; 2 Department of Organic Chemistry, Bioorganic Chemistry and Biotechnology, Faculty of Chemistry, Silesian University of Technology, Krzywoustego 4, Gliwice, 44-100 Poland; 3 Biotechnology Center, Silesian University of Technology, Krzywoustego 8, Gliwice, 44-100 Poland

**Keywords:** Atherosclerosis, Autophagy, Drug delivery, Nanogel, Trehalose

## Abstract

Atherosclerosis, cholesterol-driven plaque formation in arteries, is a complex multicellular disease which is a leading cause of vascular diseases. During the progression of atherosclerosis, the autophagic function is impaired, resulting in lipid accumulation-mediated foam cell formation. The stimulation of autophagy is crucial for the recovery of cellular recycling process. One of the potential autophagy inducers is trehalose, a naturally occurring non-reducing disaccharide. However, trehalose has poor bioavailability due to its hydrophilic nature which results in poor penetration through cell membranes. To enhance its bioavailability, we developed trehalose-releasing nanogels (TNG) for the treatment of atherosclerosis. The nanogels were fabricated through copolymerization of 6-*O*-acryloyl-trehalose with the selected acrylamide-type monomers affording a high trehalose conjugation (~ 58%, w/w). TNG showed a relatively small hydrodynamic diameter (d_H_, 67 nm) and a uniform spherical shape and were characterized by negative ζ potential (-18 mV). Thanks to the trehalose-rich content, TNG demonstrated excellent colloidal stability in biological media containing serum and were non-hemolytic to red blood cells. In vitro study confirmed that TNG could stimulate autophagy in foam cells and enhance lipid efflux and in vivo study in ApoE^−/−^ mice indicated a significant reduction in atherosclerotic plaques, while increasing autophagic markers. In conclusion, TNG hold great promise as a trehalose delivery system to restore impaired autophagy-mediated lipid efflux in atherosclerosis and subsequently reduce atherosclerotic plaques.

**Graphical abstract:**

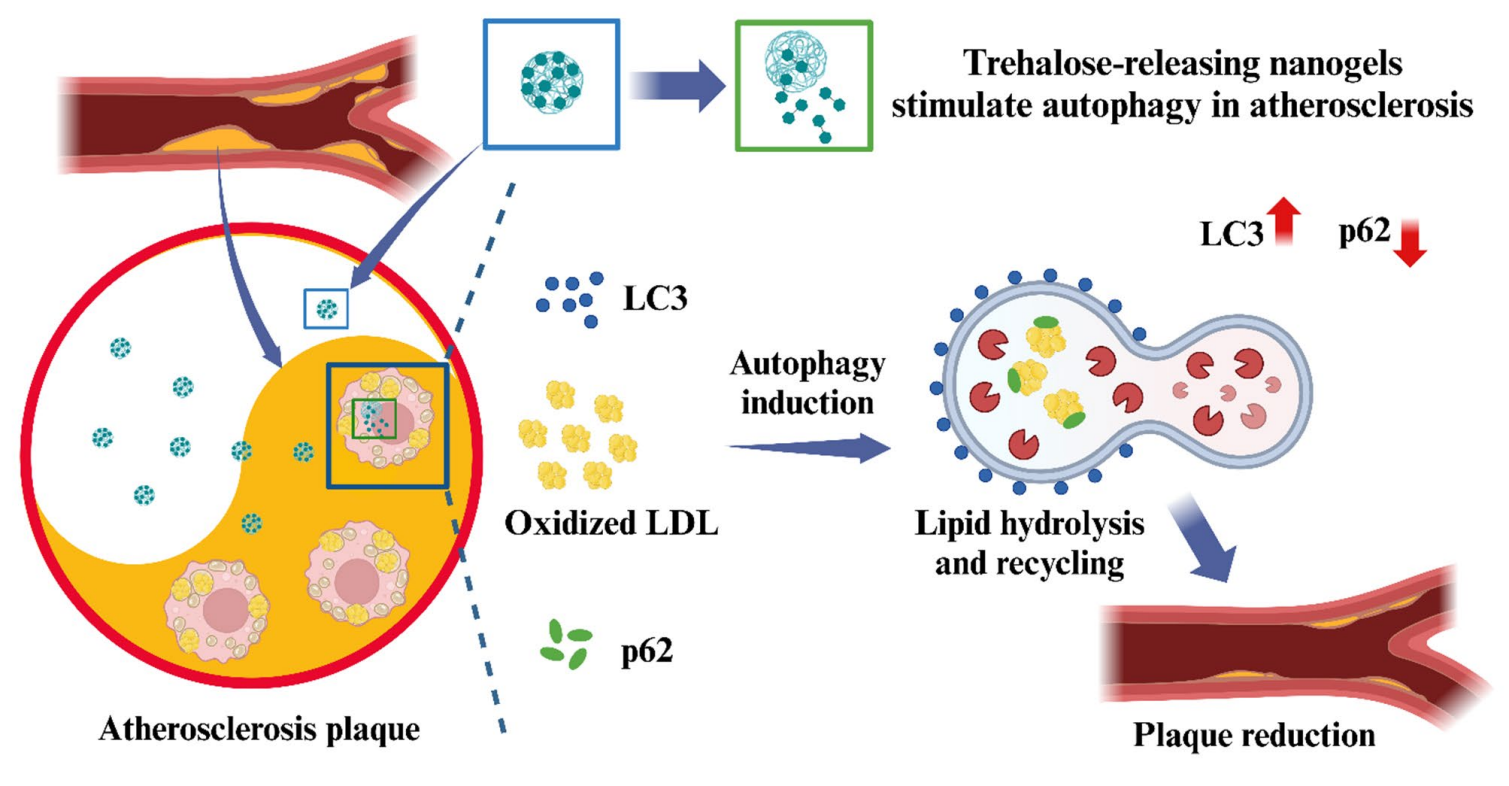

**Supplementary Information:**

The online version contains supplementary material available at 10.1186/s12951-023-02248-9.

## **Introduction**

Atherosclerosis, commonly referred to as the “silent killer,” is a chronic disease involving lipid deposition-mediated inflammation in arterial walls that builds up plaques and causes narrowing blood vessels. The pathological inflammation of atherosclerosis is complex, where it involves different cells and cellular mechanisms [1]. During atherosclerosis progression, the cellular autophagy mechanism, i.e., cellular recycling process, is impaired due to the excessive reactive oxygen species (ROS) generation-mediated oxidative stress [2, 3]. Autophagy is essential in early and mid atherogenesis (fatty streak and intermediate lesion formation), especially for lipid recycles [4–6]. Studies have shown that autophagy stimulation in macrophage-derived foam cells decelerated plaque development by enhancing cholesterol efflux [4–6]. Meanwhile, in advanced lesions, autophagy showed a protective role in maintaining plaque stabilization by promoting macrophage survival [7]. Autophagy is important in atherosclerosis not only in macrophages but also in smooth muscle cells and endothelial cells. The activation of autophagy in atherosclerotic smooth muscle cells maintains the cell phenotype and cell survival, which prevent smooth muscle cells transformation into synthetic, macrophage-like, and osteochondrogenic phenotypes [8]. Meanwhile, endothelial autophagy maintains cell alignment and protects blood vessels from shear stress-mediated atherosclerotic plaque formation by preventing endothelial apoptosis, senescence, and inflammation [9]. Taken together, the activation and recovery of autophagy as an intracellular degradation process is required in all stages of atherogenesis.

Among therapeutic drugs associated with cardiovascular disease and atherosclerosis treatments, lipid-lowering agents (e.g., statins and their derivatives) are the most commonly used due to their ability to support lowering low-density lipoprotein cholesterol (LDL-c), but they still showed adverse effects in some patients (< 5%), which are particularly related to muscle symptoms [10]. Aspirin, on the other hand, is used as anti-platelet medication to reduce the risk of blood clot formation-mediated thrombosis in atherosclerotic plaques, but not reducing the plaques [11]. Another commonly used drug is rapamycin and its derivatives as mTOR inhibitors, which are beneficial to suppress cell proliferation and promote autophagy, thereby reducing the plaques [12]. However, rapamycin has poor solubility and it might promote some adverse effects, such as hyperlipidemia and delayed re-endothelization in patients after stent angioplasty [13]. Recently, α,α-trehalose, an FDA-approved water-soluble autophagy inducer has been comprehensively studied for the treatment of impaired-autophagy related diseases (e.g., neurodegenerative disorders, diabetes and fatty liver diseases, and ischemic-related diseases) [14–19]. Trehalose has also been proved to be an effective autophagy inducer in atherosclerosis, which drives macrophage autophagy-lysosomal biogenesis [20–23], thus it is beneficial for a long-term treatment to diminish the accumulated lipids in the plaques. Trehalose, however, has low bioavailability that requires the use of relatively high concentrations to induce autophagy in cells [24, 25]. In animal studies, trehalose is usually administered intraperitoneally at the dose of 2–3 g/kg or by oral administration in drinking water (2–4% w/v) [24–27]. The hydrophilic nature of trehalose results in poor penetration through cell membranes. In addition, the presence of trehalase enzyme in the intestinal mucosa affects its pharmacokinetic behavior due to rapid hydrolysis. Therefore, there is a need for seeking effective trehalose delivery systems using nanocarriers, which could enhance the bioavailability of trehalose, as well as deliver trehalose to atherosclerotic plaques for stimulating autophagy-mediated recovery of cellular recycling process.

Although a number of studies have explored the use of nanoparticles, particularly smart nanocarriers, for delivering ROS scavenging agents, matrix metalloproteinase (MMP) inhibitors, lipid-lowering agents, and photo thermal and photodynamic activating agents for atherosclerosis treatment, only few studies have focused on specifically restoring autophagy in atherosclerosis [28]. Very recently, three significant reports in this topic have been published. Firstly, Guo et al. (2022) reported the use of self-assembled LOX1-targeted siRNAs and conjugated rapamycin/DNA nanomicelles for stimulating autophagy in atherosclerosis. This approach was found to mediate lipid clearance and prevent the formation of foam cells by restoring the cellular recycling process [29]. Secondly, You et al. (2022) used atorvastatin-loaded graphene oxide quantum dots (GOQDs) modified with hybrid cell membrane coating for enhanced atherosclerosis targeting and autophagy stimulation [30]. Lastly, the first use of nanocarrier-based trehalose as an autophagy inducer was introduced by Wu et al. (2022) [31]. They developed self-assembled trehalose-bearing arginine and phosphatidylserine as nanomotors by accelerating penetration of nanomotors to target macrophages using nitric oxide (NO) as the driving force, which was generated from the reaction between arginine and ROS in the interstitial fluid of atherosclerotic lesions. Trehalose nanomotors-mediated autophagy activation in a mice model of atherosclerosis led to a significant reduction in macrophage foam cell formation, improvement in endothelial cell integrity, and most notably, a decrease in the lesion area [31]. Stimulation of autophagy to treat atherosclerosis is also targeted in the study from Li et al., who developed multi-loaded self-assembled nanovesicle systems composed of amphiphilic H9 peptide and hexadecyl phosphorylcholine loaded with physically entrapped trehalose and the (HP-β-CD)/oridonin inclusion complex [32]. The synergistic effects of oridonin and trehalose could inhibit foam cell formation in RAW264.7 cells, reduce inflammatory cytokines IL-1β, IL-6, and TNF-α, and promote the formation of autophagosomes, as confirmed by the increased level of LC3 in foam cells.

**Fig. 1:**
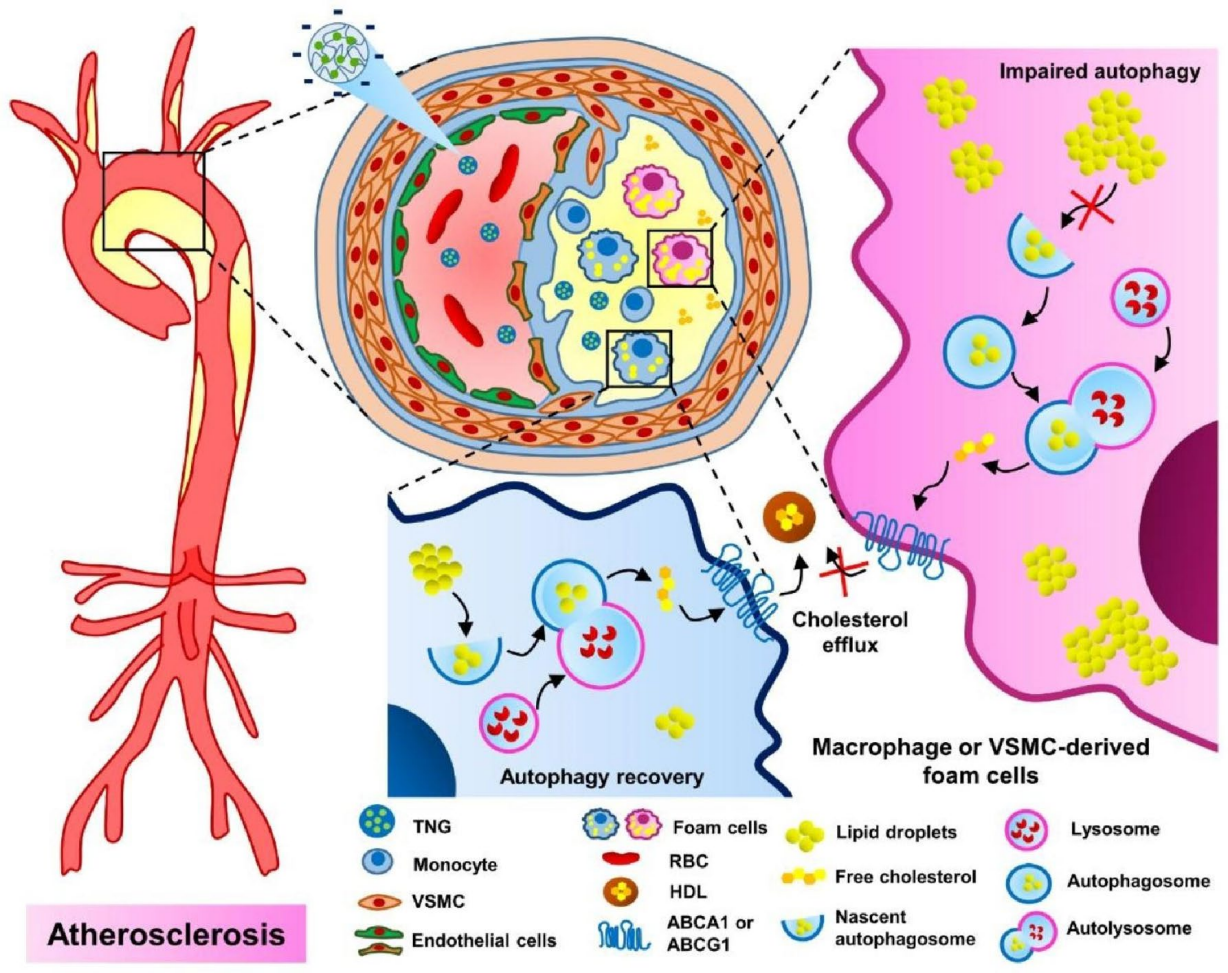
Scheme of the hypothesis of how trehalose-releasing nanogels (TNG) could promote atherosclerosis management via the autophagy recovery-mediated lipid recycling process and cholesterol efflux. TNG: trehalose-releasing nanogels, VSMC: vascular smooth muscle cell, RBC: red blood cell, HDL: high density lipoprotein, ABCA1: ATP Binding Cassette Subfamily A Member 1, ABCG1: ATP Binding Cassette Subfamily G Member 1

Herein, we designed nanogels with covalently bound, and releasable trehalose for autophagy stimulation in atherosclerosis. Specifically, trehalose was incorporated within nanogels in free radical copolymerization through its 6-*O*-acryloyl derivative, affording nanogels with high trehalose conjugation (~ 58%, w/w). In order to achieve controlled release of trehalose under physiological conditions, we co-incorporated acrylamide (AM), which facilitates hydrolysis of the ester bond in 6-*O*-acryloyl-trehalose units, thus free trehalose can be sustainably released from the nanogel network. Based on our preliminary study [33], a significant increase in trehalose release was noticed in the nanogels with high AM content in comparison to those without AM. We also found that high content of trehalose is beneficial for the colloidal stability in biological media, especially in serum-containing media [33]. More importantly, in vivo studies in transgenic *Drosophila* and zebrafish larvae showed a significant induction of autophagy after treatment with trehalose-releasing nanogels [33]. In the current study, we used a mice model of atherosclerosis to observe the efficacy of TNG for potential nanotherapy of atherosclerosis via autophagy modulation (Fig. 1).

## Results and Discussion

### Synthesis and characterization of trehalose-releasing nanogels

Nanogels, i.e., nanoparticles based on crosslinked hydrophilic polymeric networks, are among the most promising and widely studied nanocarriers as drug delivery systems due to their characteristics, such as high biocompatibility, high loading capacity, potential biodegradability and responsivity to biological cues [34]. Nanogels were first introduced and applied as nanocarriers for the delivery of antisense oligonucleotides by Kabanov’s group in 1999 [35], following massive studies.

**Fig. 2:**
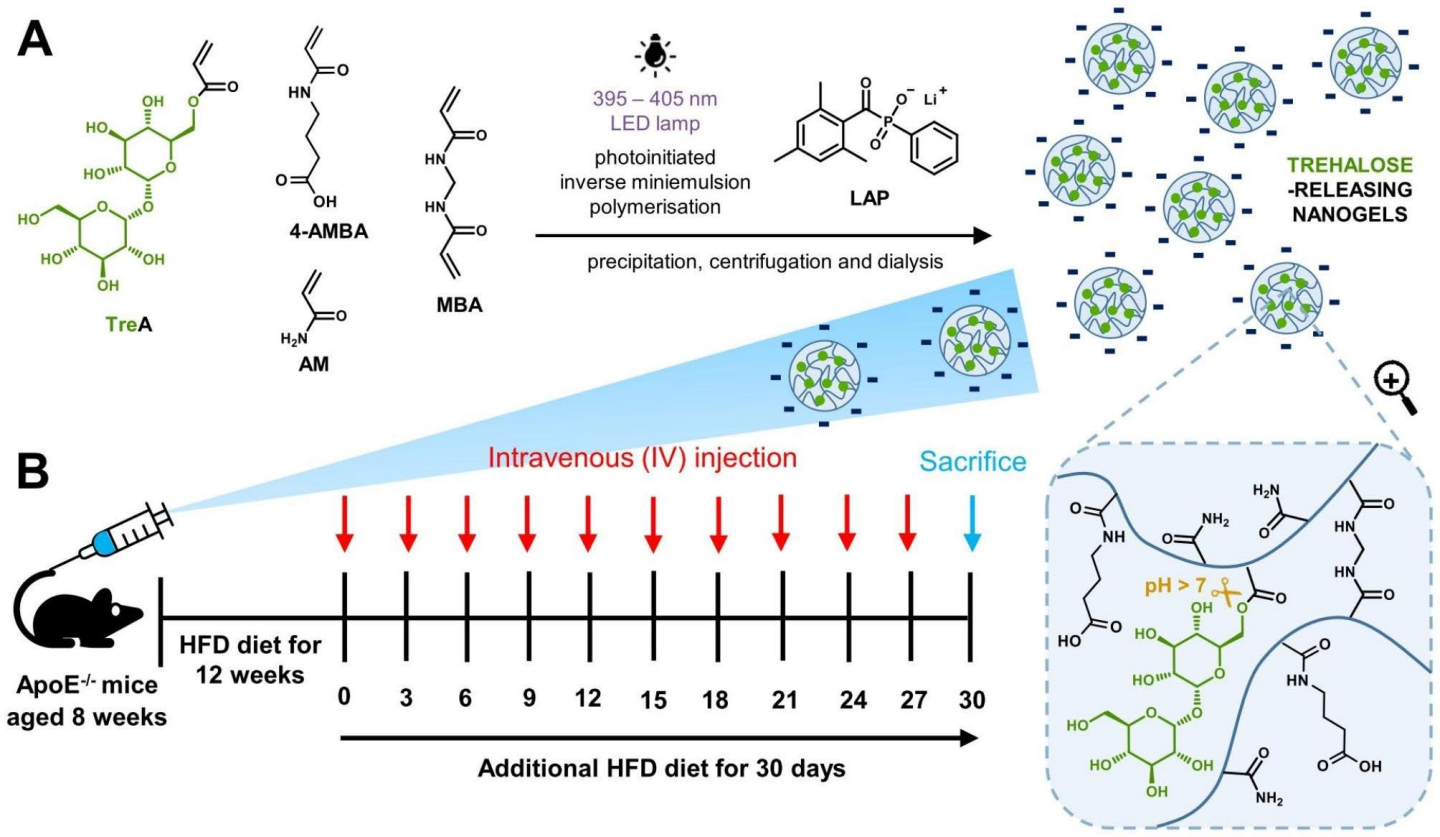
(**A**) The synthesis of trehalose-releasing nanogels (TNG), and (**B**) timeline for ApoE^−/−^ mice pretreatment and systemic administration of TNG via the tail vein

**Fig. 3:**
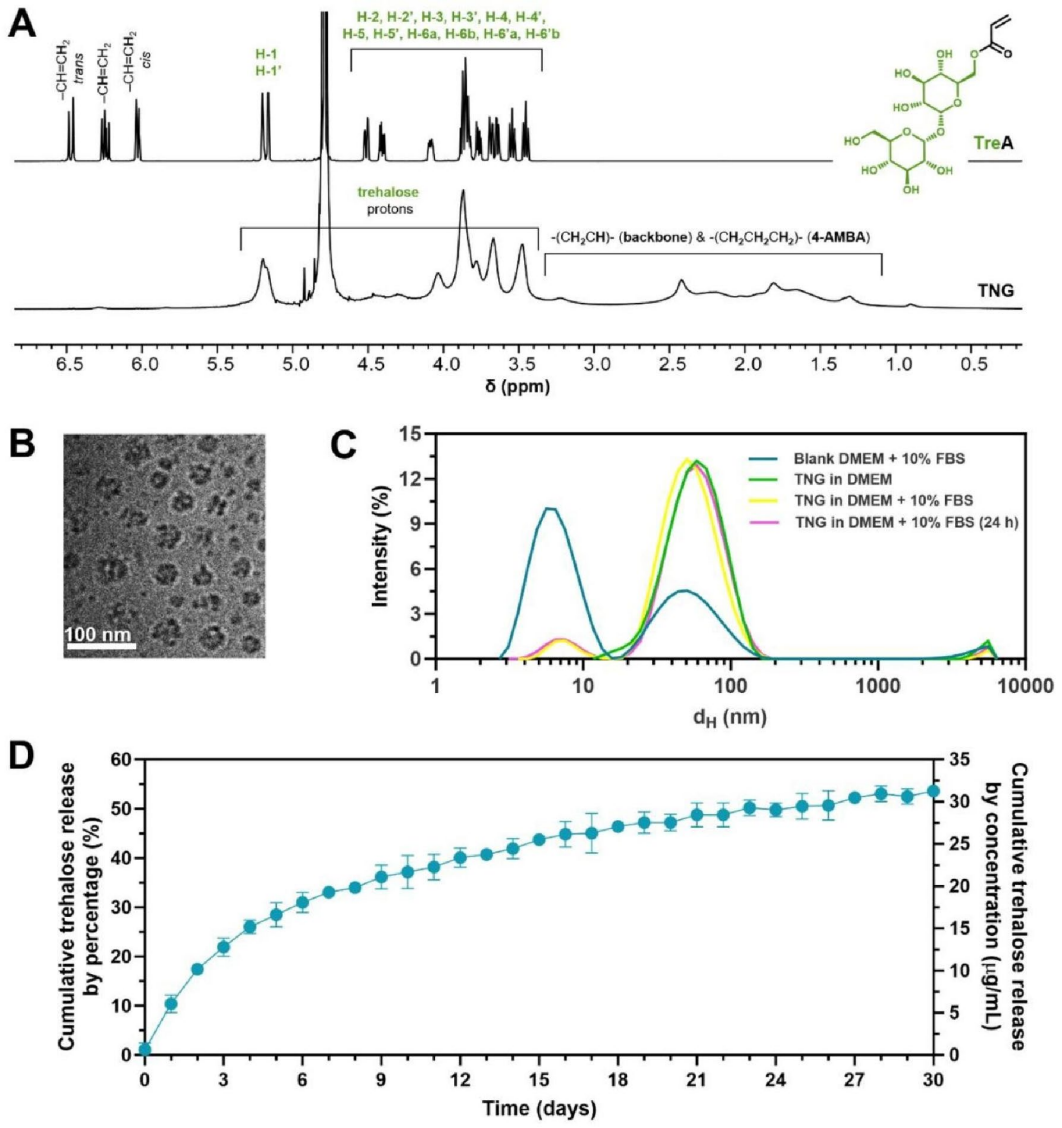
Characterization of trehalose-releasing nanogels (TNG). (**A**) Section of ^1^H NMR spectra of TreA (top) and TNG (bottom) proving covalent incorporation of trehalose into TNG (D_2_O, 600 MHz). (**B**) Cryo-TEM micrograph of TNG in water. Scale bar: 100 nm. (**C**) Colloidal stability of TNG in DMEM + 10% FBS monitored by DLS measurement. (**D**) Trehalose release profile from TNG in PBS over one month (pH 7.4, 37 °C, TNG concentration: 100 µg/mL). Data are presented as mean ± SD, *n* = 3

In the current study, we designed nanogels with covalently bound and releasable trehalose, denoted as TNG, to treat atherosclerosis via autophagy recovery. Trehalose was incorporated within nanogels in free radical copolymerization through its 6-*O*-acryloyl derivative, yielding trehalose-rich nanogels with ~ 58% covalently bound trehalose. AM-type monomers were used to enhance the release of trehalose through ester bond hydrolysis at physiological conditions (Fig. 2A). As demonstrated previously, copolymerizing 6-*O*-acryloyl-trehalose with acrylamide-type monomers allows to fabricate materials that can sustainably release trehalose at pH 7.4 due to the interaction of the amide protons with the ester bond in adjacent acrylate units, which strongly accelerates ester hydrolysis [36, 37]. The presence of covalently bound trehalose and the purity of TNG was confirmed by ^1^H NMR spectroscopy (Fig. 3A). The presence of broad signals typical for polymers in the range of 3.3–4.6 and 5.0-5.5 ppm, which are well correlated with proton signals of the trehalose monomer TreA, and the lack of signals from protons of the acrylate group in the range of 6.0–6.5 ppm, both prove successful incorporation of TreA into the TNG network. Signals originating from the protons of other key structural fragments, e.g., methylene groups from 4-AMBA (1.7–1.9, 2.3–2.5 and 3.1–3.3 ppm), and polymer backbone derived from acrylates and acrylamides (1.1–3.0 ppm) can also be easily identified.

Based on our previous study [33], we noticed that an improved colloidal stability of nanogels in biological media was ensured by incorporating ionic monomer or high content of trehalose in the nanogel network. Trehalose seemed to have a significant role in improving the colloidal stability of nanogels especially in biological media, and particularly in serum-containing media. Thereby, to further prove the beneficial effect of trehalose on nanogel colloidal stability, in the current study we tested whether by replacing TreA monomer with 2-hydroxyethyl acrylate (HEA) monomer (hydrophilic monomer with one hydroxyl group) had a significant impact on the colloidal stability of AM-based nanogels. HEA-containing nanogels were synthesized by substituting TreA with HEA at both equimolar and equimass of TreA feed (Table S1) for obtaining HEA_1_NG and HEA_2_NG, respectively. Physicochemical characteristic of nanogels is shown in Table 1. The yield after polymerization of TNG, HEA_1_NG, and HEA_2_NG were 67, 75, and 69%, respectively.

**Table 1:** Physicochemical characterization of nanogels

Sample	Trehalose conjugation (% w/w)	Yield (%)	Z-average d_H_ (nm) (PdI)	ζ potential (mV)
TNG	57.6	67	67 (0.277)	-17.6 ± 6.1
HEA_1_NG	-	75	78 (0.283)	-18.9 ± 5.3
HEA_2_NG	-	69	80 (0.230)	-19.4 ± 5.9

TNG - trehalose-releasing nanogels

HEA_1_NG and HEA_2_NG − 2-hydroxyethyl acrylate-based nanogels

From DLS measurement, the Z-average d_H_ and PdI of TNG, HEA_1_NG, and HEA_2_NG were 67, 78, and 80 nm and 0.277, 0.283, and 0.230, respectively. All nanogels were also characterized by a similar ζ potential of about − 18 mV (Table 1). Cryo-TEM image showed that the diameter of TNG was ~ 50 nm (Fig. 3B). The colloidal stability of nanogels was assessed by monitoring the transmittance in various biological media over time. The transmittance of TNG dispersions in water, PBS (pH 7.4), normal saline (NS), DMEM, and DMEM + 10% FBS at high concentration of 1.0 mg/mL within 7 days of incubation at 37 ºC, exceeded 95%, which indicated their excellent stability (Figure S1). In contrast, both HEA_1_NG, and HEA_2_NG were only stable in water but they aggregated immediately in PBS (pH 7.4), NS, DMEM, and the most aggregation was found in DMEM + 10% FBS. It is possible that the hydrophilic nature of pendant trehalose may have a huge impact on their colloidal stability due to short-range repulsive hydration forces [38]. The aggregation of nanoparticles in biological media is not preferred both for in vitro and in vivo study, which might result in cytotoxicity profile, low cellular uptake, misleading of specific targeting, and elimination by endothelial reticular system (RES) during nanoparticle circulation [39–41]. Further colloidal stability study was conducted by DLS to observe the stability of TNG in DMEM + 10% serum. The result showed that the size distribution of TNG in DMEM + 10% FBS remained stable within 24 h of incubation at 37 ºC (Fig. 3C), which was plausible for preparation of intravenous (*i.v.*) injection. The amount of trehalose incorporated into TNG was determined enzymatically after pretreating nanogel with strong alkali. Under these conditions all trehalose was cleaved into solution. From the enzymatic assay, the content of trehalose in TNG was around 58% (w/w) (Table 1, Figure S3). The release study showed that trehalose could be sustainably released at pH 7.4, 37 ºC, reaching nearly 55% release within 30 days, what corresponds to about 32 µg/mL of trehalose (at a total trehalose concentration of ~ 58 µg/mL) (Fig. 3D). The sustained release of trehalose from TNG would be desirable for in vivo study of atherosclerosis treatment within one month (Fig. 2B).

### Biocompatibility of trehalose-releasing nanogels

Red blood cell compatibility is crucial to ensure that nanocarriers or nanoparticles do not cause hemolysis (rupture of red blood cells) or other adverse effects when interacting with red blood cells [42]. For the in vivo drug delivery system, ensuring good blood compatibility is essential, along with low toxicity and appropriate nano-size. The evaluation of hemolytic activity of the administered nanogel is crucial to guarantee safety during administration. As seen in Figure S4, the results demonstrated that all tested samples showed no significant hemolysis, even at a very high concentration of 2 mg/mL, which was extremely higher than the practical concentration in the bloodstream. Although there was a slight increase in hemolysis compared to free trehalose, the hemolysis ratios of TNG remained below 5%. This percentage is generally considered safe for in vivo applications, according to the criterion outlined in the ASTM E2524-08 standard [42]. The standard states that materials with a hemolysis percent higher than 5% pose a risk of damaging red blood cells during in vivo applications. Therefore, the evaluated nanogel was demonstrated to be a favorable blood compatibility and met the safety requirements for subsequent in vivo application.

The cytotoxicity profile of TNG at different concentrations was also examined in the cultured HUVECs for 24 h. The results indicated that TNG at reasonable in vitro doses (≤ 200 µg/mL) did not cause cytotoxic effects in HUVECs (more than 90% cell viability) (Figure S5), indicating the biocompatibility of TNG for further in vitro studies.

Due to the hydrophilicity of trehalose, its ability to penetrate cell membranes is limited, hindering its bioavailability. To improve the membrane permeability and bioavailability of trehalose, the cellular uptake capacity of TNG was evaluated. The fluorescence intensity of TNG group was significantly higher than that of free trehalose group (Figure S6). Such results suggest that formulation manipulation may be a viable avenue to improve the pharmacokinetics of drugs.

### In vitro assessment of autophagy stimulation and anti-atherosclerosis effects by trehalose-releasing nanogels

**Fig. 4:**
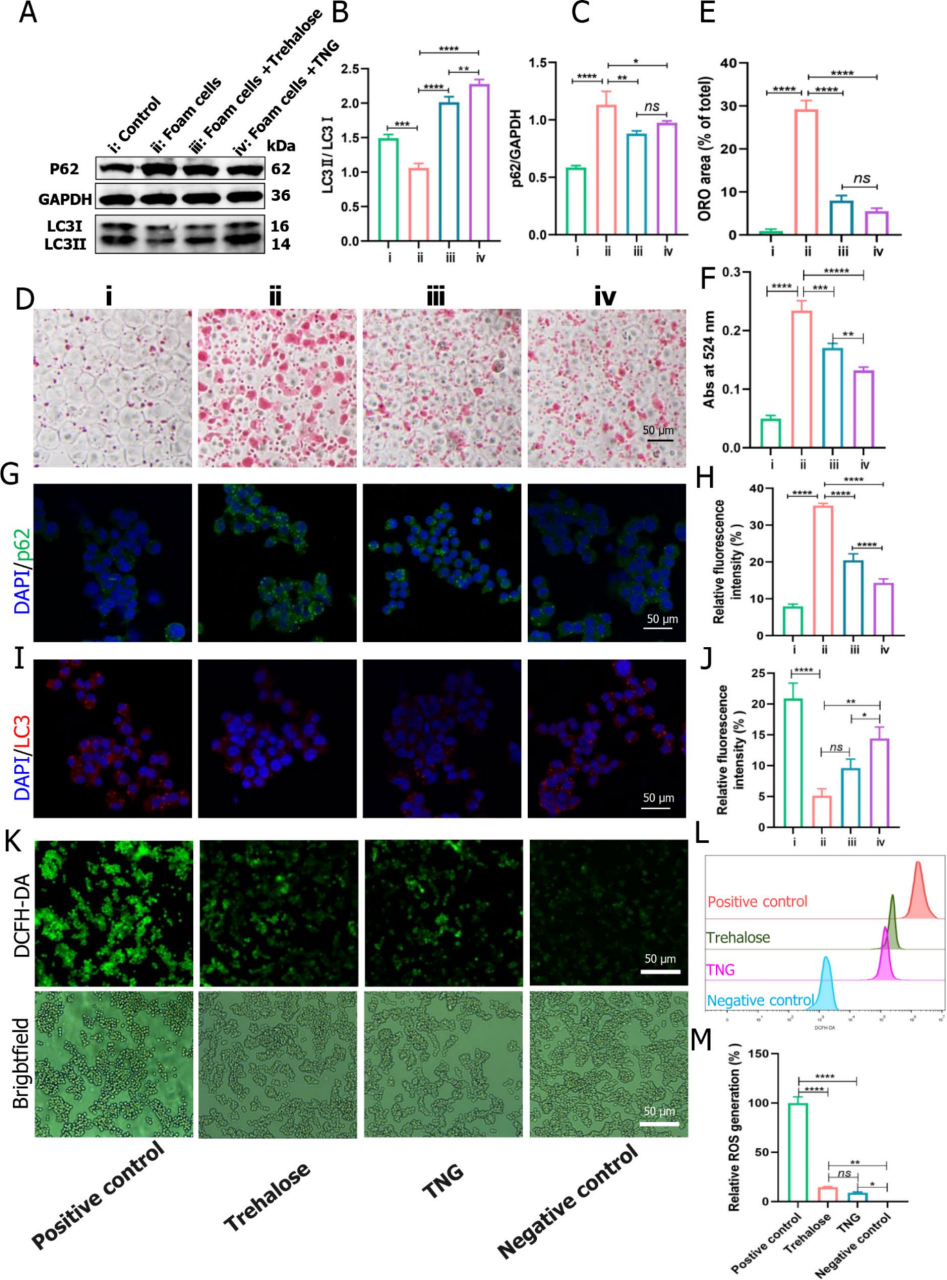
In vitro autophagy stimulation and anti-atherosclerosis effects. (**A**) Western blot analysis of p62, LC3-I, LC3-II, and GAPDH protein expressions from foam cells treated with or without free trehalose and nanogel in comparison to the control); i = control, ii = foam cells, iii = foam cells + trehalose, and iv = foam cells + TNG. (**B, C**) Quantification of LC3-II/LC3-I and p62/GAPDH ratios from the Western Blot images. (**D**) Lipid profile in foam cells treated with or without free trehalose and nanogel in comparison to the control upon oil Red O (ORO) staining, following quantification of (**E**) lipid droplet area (% of total) and (**F**) Abs at 524 nm; i = control, ii = foam cells, iii = foam cells + trehalose, and iv = foam cells + TNG. (**G, I**) Fluorescence imaging and (**H, J**) quantification of p62 and LC3 expressions from foam cells treated with or without free trehalose and TNG in comparison to the control. (**K**) Fluorescence images and (**L, M**) quantification by flow cytometry showing intracellular ROS generation after treatment with different formulations and stimulation with H_2_O_2_. RAW264.7 cells were incubated with medium alone or trehalose or TNG for 4 h, followed by stimulation with H_2_O_2_ for 0.5 h. Cells unstimulated with H_2_O_2_ served as the negative control. Then fluorescence microscope and flow cytometric analyses were performed. Scale bar: 50 μm. Data are presented as mean ± *SD*, *n* = 3, *ns*: no significance, *p < 0.05, **p < 0.01, ***p < 0.001, and ****p < 0.0001

The in vitro autophagy stimulation and anti-atherosclerosis effects were assessed in macrophage-derived foam cells. The relationship between autophagy and LC3 is crucial in understanding the process of autophagy at the molecular level. LC3, specifically LC3-I (cytosolic form) and LC3-II (lipidated form), is an essential component of the autophagosomal membrane and plays a central role in autophagosome formation and maturation. In the in vitro study, macrophage-derived foam cells exhibited a decreased level of LC3 but an elevated level of p62 compared to the control group, as evidenced by Western blot analysis (Fig. 4A-C). However, upon treatment with trehalose and TNG, there was a significant increase in the LC3-II/LC3-I ratio, accompanied by a decrease in the p62/GAPDH ratio. These findings indicated that trehalose and TNG induced autophagy in the foam cells. Moreover, fluorescence analysis yielded similar results, showing a significant reduction in the p62 level in foam cells, which could be attributed to the degradation of p62 during the autophagy process (Fig. 4G, H). The activation of autophagy in atherosclerosis results in the interaction between p62 and autophagy substrates, forming a structure known as phagophore, which involves the participation of LC3 (conversion from LC3-I to LC3-II) [43, 44]. LC3-II acts as a key adaptor molecule in autophagy by binding to various autophagy-related proteins and interacting with cargo molecules, such as damaged proteins and organelles, to be sequestered into the autophagosome. This selective engulfment of cargo is essential for autophagy to degrade and recycle cellular components properly. The level of LC3-II is often used as a marker to monitor autophagic activity. Typically, there is a strong correlation between the upregulation of LC3-II expression relative to LC3-I and the downregulation of p62 expression, which indicate an enhancement in autophagic flux. Meanwhile, there was a significant increase in the LC3 fluorescence compared to the negative control, indicating that the autophagy was recovered (Fig. 4I, J). The absorbance of Oil Red O (ORO)-stained foam cells, subjected to free trehalose and TNG treatments, exhibited a reduction compared to untreated foam cells (Fig. 4D-F). Remarkably, TNG treatment manifested the most pronounced efficacy, furnishing additional substantiation of its in vivo anti-atherosclerotic potential. To further investigate the ROS scavenging performance, TNG and trehalose were used to treat the RAW264.7 cells with the addition of H_2_O_2_. In the positive control group, RAW264.7 cells were simultaneously treated with 0.5 µM H_2_O_2_, while cells cultured with medium alone served as the negative control. After stimulation for 0.5 h and incubation with free medium for the additional 2 h, cells in the positive control displayed a considerably high level of ROS, as probed by a fluorescent dye 2’,7’-dichlorofluorescin-diacetate (DCFH-DA) that emits green fluorescence under oxidative conditions (Fig. 4K). In contrast, fluorescent signals of DCFH-DA were dramatically decreased when activated macrophages were treated with free trehalose or TNG for 4 h. Further quantitative analysis by flow cytometry also demonstrated that the intracellular ROS production in stimulated macrophages could be effectively suppressed by treatment with TNG (Fig. 4L, M).

### In vivo plaque targeting and biodistribution study of trehalose-releasing nanogels

**Fig. 5:**
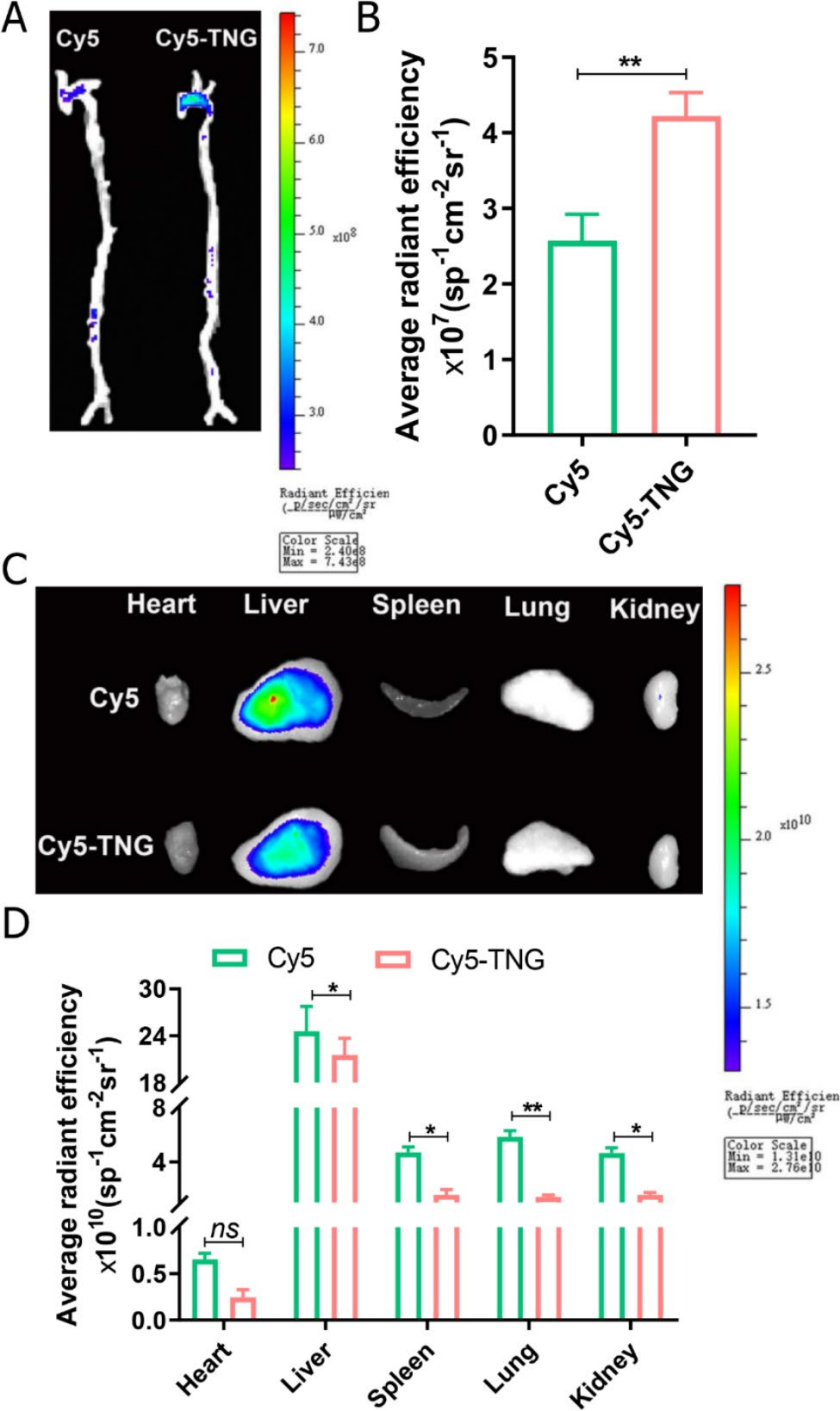
In vivo plaque targeting and biodistribution of trehalose-releasing nanogels in major organs. (**A**) The ex vivo fluorescence images of the aorta and (**B**) quantitative analysis of fluorescence signals accumulated in the aorta of ApoE^−/−^ mice after 24 h of IV with Cy5-TNG and free Cy5. (**C, D**) The ex vivo biodistribution images of Cy5-TNG and free Cy5 in major organs (heart, liver, spleen, lung, and kidney). Data are presented as mean ± *SD*, *n* = 3, *ns*: no significance, *p < 0.05 and **p < 0.01

To investigate the in vivo pharmacokinetic profile of TNG in C57BL/6 mice, after *i.v.* injection of Cy5-labeled TNG (Cy5-TNG) and free Cy5, the results indicated that Cy5 was gradually decreased and almost completely cleared out from blood until 24 h, while the fluorescence intensity of Cy5-TNG was nearly 30% of the initial one at the end of investigation (Figure S7). Then we investigated in vivo targeting capability of TNG in apolipoprotein E-deficient (ApoE^−/−^) mice. The Cy5-TNG and free Cy5 were administered through *i.v.* administration, and let them circulating in the bloodstream for a duration of 24 h. As illuminated by the results of fluorescent imaging, Cy5-TNG exhibited a conspicuous propensity for accumulation within aortic plaques, surpassing the observed accumulation of free Cy5, with an average Region of Interest (ROI) approximately 1.6 times that of free Cy5 (Fig. 5A, B). Additionally, in relation to other organs (heart, spleen, lung, and kidney), both Cy5-TNG and free Cy5 primarily accumulated in liver (Fig. 5C, D), given the liver’s prominence as the body’s largest metabolic organ. The results imply that Cy5-TNG possesses a preferential affinity for targeting plaques and manifest an extended blood half-life.

### In vivo assessment of autophagy stimulation and anti-atherosclerosis effects by trehalose-releasing nanogels

**Fig. 6:**
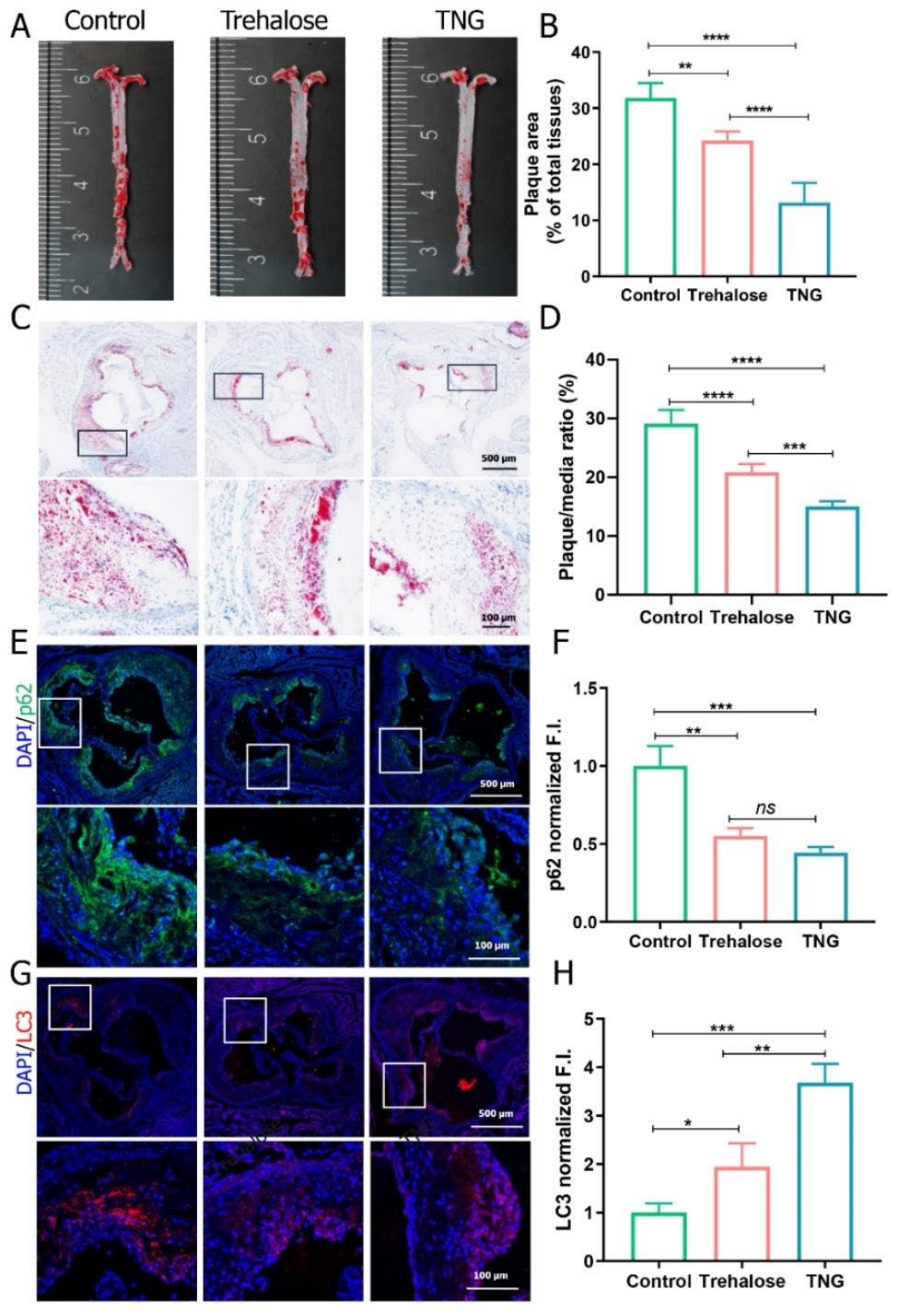
In vivo autophagy stimulation and anti-atherosclerosis effects of trehalose-releasing nanogels. (**A**) The en face oil red O (ORO)-stained images of aortas in ApoE^−/−^ mice after one month of treatments with free trehalose and TNG compared to the control and (**B**) quantitative analysis of the lesion area. (**C**) ORO-stained images of aortic root sections in ApoE^−/−^ mice after one month of treatments with free trehalose and TNG compared to the control and (**D**) quantitative analysis of the atherosclerotic plaque area. (**E, G**) CLSM images and (**F, H**) quantitative analysis of p62 (green fluorescence) and LC3 (red fluorescence) expressions in atherosclerotic plaques of the aortic root sections in ApoE^−/−^ mice after one month of treatments with free trehalose and TNG compared to the control, scale bars: 500 and 100 μm, respectively. Data are presented as mean ± *SD*, (**A–D**) *n* = 5, (**E–H**) *n* = 3, *ns*: no significance, *p < 0.05, **p < 0.01, ***p < 0.001, and ****p < 0.0001

After testing the ability of TNG in stimulating autophagy and lipid efflux in foam cells, we tested its effects in vivo. TNG and free trehalose were administered intravenously every three days, for one month, to ApoE^−/−^ mice that had been treated with HFD for 12 weeks. The findings indicated that TNG exhibited a significant anti-atherosclerosis effect, leading to a reduction of approximately 60% in the overall plaque area after one month of treatments. Free trehalose was also confirmed to have the ability to decrease the total plaque area but to a lesser extent compared to TNG (Fig. 6A–D). This gap may be related to the poor bioavailability and disappointing pharmacokinetics of trehalose.

To assess the enhanced autophagy effect of TNG, we analyzed the p62 and LC3 expressions in the atherosclerotic plaque area using immunofluorescent staining. The findings revealed that mice treated with TNG exhibited a significant decrease in p62 levels, surpassing 50% reduction, and a nearly four-fold increase in LC3 levels. Similarly, the administration of free trehalose could promote autophagy in atherosclerosis, as evidenced by the reduction in p62 levels and the elevation of LC3 levels (Fig. 6E –H). However, its effect on inducing autophagy was not as pronounced as that of TNG. These were consistent with the results of previous in vitro investigations. In the management of atherosclerosis, trehalose could activate TFEB, leading to the successful recovery of damaged autophagy in plaque. This repair process enhanced the elimination of p62-enriched aggregates and the expression of various components related to autophagy (such as LC3), which was conducive to the formation of autophagosomes [45]. Separate staining with anti-CD68 antibody indicated that TNG treatment effectively reduced the macrophage count in plaques (Figure S8A, B). As the degree of macrophage infiltration is positively correlated with plaque vulnerability, these findings indicate that TNG therapy has the potential to enhance the stability of atherosclerotic plaques and impede the progression of atherosclerosis more efficiently than free trehalose. It is surprising that, despite the important role of trehalose in restoring autophagy, there have been only a few studies focused on the development of nanocarriers to deliver trehalose to the atherosclerotic plaques. In addition, due to trehalose’s high hydrophilicity and difficulty in crossing phospholipid bilayer of cell membranes [26, 27], the developments of nanocarriers that can deliver trehalose intracellularly are paramount important, particularly for atherosclerosis management. In the present work, we have demonstrated that our developed nanogels could significantly outperform free trehalose in promoting lipid removal, reducing plaque area, and efficiently enhancing autophagy in atherosclerosis.

### In vivo biosafety assessment of trehalose-releasing nanogels

In the end of study, we examined the biosafety assessment of free trehalose and TNG compared to the control, which included H&E staining of the main organs, body weight, CBC profile, and blood lipid profile (Figure S9A − E, Tables S2–S4). The tissue morphology of the mice treated with free trehalose and TNG did not differ from the control group, and the liver and kidney function indexes fell within the normal reference range. TNG demonstrated the best lipid profile in comparison to the control group, with considerably lower levels of total cholesterol and LDL-C and higher levels of HDL-C. In addition, we observed fluctuations in serum of alanine aminotransferase (ALT), aspartate aminotransferase (AST), creatinine (CR) and uric acid (UA) but were within the normal reference range.

## Conclusion

We have successfully developed trehalose-releasing nanogels (TNG) using a facile synthesis method through photo-initiated free radical polymerization and mini-emulsion technique, yielding nanogels with exceptionally high content of covalently bound trehalose (~ 58%). TNG had a uniformly spherical shape with the average size of ~ 50 and 67 nm (d_TEM_ and d_H_, respectively), and had a negative surface charge. Most importantly, TNG were capable of releasing trehalose in physiologically relevant conditions (pH 7.4, 37 °C) through the cleavage of trehalose-conjugating ester bonds facilitated by the co-presence of acrylamide-type units. Thanks to the trehalose-rich content, TNG were very stable in serum-enriched media and did not hemolyze red blood cells even at relatively high concentrations. Moreover, TNG were able to accumulate in atherosclerotic plaques with low accumulations in the main organs except liver. Finally, in vitro and in vivo studies in atherosclerosis confirmed that TNG could enhance the autophagy process, facilitate lipid removal, and reduce plaque area by approximately 60%. Taken together, TNG are promising trehalose delivery systems for atherosclerosis management.

## Materials and Methods

### General methods

Ultrasonication with Sonics VCX 130 (diameter of probe: 3 mm, Sonics & Materials, Inc., USA) was carried out during preparation of a miniemulsion (before photo-polymerization) and redispersion of nanogel powder at amplitudes of 60% (5 min) and 40% (30 s), respectively. Each purification process was followed by dialysis and lyophilization in a freeze dryer (ALPHA 1–2 LDplus, CHRIST). NMR spectra were recorded in deuterated solvents (Deutero GmbH) with internal standards using an NMR spectrometer operating at 600 MHz (Varian). Fluorescence study was conducted in SpectraMax i3x Multi-Mode Microplate Reader (Molecular Devices, USA). Both the amount of conjugated trehalose and trehalose release profile were determined enzymatically using a Trehalose Assay Kit (Megazyme International, Ireland). Phosphate buffered saline (PBS, pH 7.0 and 7.4), potassium chloride (KCl), and normal saline (NS) solutions. Deionized water (DI water) was produced using a reverse osmosis system (conductivity < 2 µS/cm).

#### Materials and reagents for synthesis of nanogels

Acrylamide (AM, Acros Organics), *N,N’*-methylenebisacrylamide (MBA, Acros Organics), 2-hydroxyethyl acrylate (HEA, Acros Organics), lithium phenyl-(2,4,6-trimethylbenzoyl) phosphinate (LAP, Carbosynth), sorbitane monooleate (Span 80, Sigma Aldrich), cyclohexane (Chempur), acetone (Chempur), sulfo-Cy5-amine (Lumiprobe), dialysis membrane (Spectrum™ Spectra/Por™ 2 RC Dialysis Membrane, MWCO: 12–14 kDa). 6-*O*-acryloyl-α,α’-trehalose (TreA) was synthesized following our previously described method [36]. 4-acrylamidobutanoic acid (4-AMBA) was synthesized according to Yaşayan et al. (2012) [46]. 4-acrylamidobutanoic acid 3-sulfo-N-hydroxysuccinimide ester sodium salt (4-AMBA-Sulfo-NHS) was synthesized based on the method reported by Tsuji et al. (2019) for the synthesis of homologous *N*-sulfosuccinyl-6-hexyloylacrylamide sodium salt [47] and the detailed synthesis procedure is provided in Supplementary Information. 

#### Synthesis of trehalose-releasing nanogels (TNG) and HEA-containing nanogels (HEA_1_NG and HEA_2_NG)

TNG were synthesized via an inverse miniemulsion free-radical polymerization (FRP) according to our previously described method [33]. Briefly, a water-in-oil (w/o) miniemulsion (1:10, v/v) was created from monomers and photoinitiator-containing aqueous phase (PBS pH 6.0, 1.0 mL) and Span 80-containing organic phase (cyclohexane, 10.0 mL). MBA (20.0 mg), TreA (152.7 mg), AM (35.3 mg), and 4-AMBA (13.4 mg) were placed in a 4-mL dark vial and dissolved in PBS (pH 6.0). The solution of LAP initiator (2.3 mg, 51 µL) was added, and the aqueous phase was then transferred into a 20-mL transparent vial containing the cold organic phase (4 ºC). The miniemulsion was prepared by ultrasonication of the mixture in ice bath (60% amplitude, 5 min). After that, the vial was covered in aluminum foil and exposed for 0.5 h to high power light-emitting diodes (LEDs, 3 W, 395–405 nm) photoirradiation from the bottom of the vial. Following a precipitation step in 40 mL of acetone, the product was centrifuged at 14,610 ×*g* for 10 min, twice rinsed with 40 mL of acetone and left to air dry overnight. In order to purify the product, the crude nanogels were suspended in DI water and dialyzed against H_3_PO_4_ solution (pH 5.0, MWCO 12–14 kDa) for 24 h with multiple media changes and DI water as the last change. The nanogel dispersion was finally freeze-dried, producing a white fluffy powder that was kept at 4 ºC until usage.

To investigate the role of trehalose in nanogel stability, HEA-based nanogels were synthesized using a similar procedure as described earlier. However, in this case, the TreA monomer was replaced with the HEA monomer in both equimolar and equimass of TreA feed (Table S1) for obtaining HEA_1_NG and HEA_2_NG, respectively.

#### Synthesis of trehalose-releasing nanogels bearing active ester (NHS-TNG)

To synthesize NHS-TNG, a similar procedure as described for the synthesis of TNG was applied with the difference that 4-AMBA-sulfo-NHS (4.0 mg, 0.011 mmol) was added and the aqueous phase was prepared in PBS neutralized with NaOH to pH 6.0. The crude NHS-TNG were kept at -20 ºC without further purification for preparation of fluorescently labeled nanogels.

#### Synthesis of Cy5-labeled trehalose-releasing nanogels (Cy5-TNG)

NHS-TNG (10.0 mg) was redispersed in DMSO (250 µL) containing sulfo-Cy5-amine (0.25 mg, 0.00033 mmol) and triethylamine (0.068 mg, 0.00067 mmol). Then the nanogel dispersion was shaken overnight in an orbital shaker (1000 rpm, 25 ºC). On the next day, the volume was adjusted to 1.0 mL with DMSO, and then the product was precipitated with 3.6 mL of acetone. The suspension was then centrifuged at 14,610 ×*g* (4 ºC, 2 min) and washed six times with acetone. Nanogel precipitate was then redispersed in 800 µL of DI water, ultrasonicated at 40% amplitude (30 s), and then dialyzed against H_3_PO_4_ solution (pH 5.0, MWCO 12–14 kDa) for 24 h with multiple media changes and DI water as the last change. The pure Cy5-TNG dispersion was finally freeze-dried to obtain fine powder and kept at 4 ºC prior to use.

#### Dynamic light scattering (DLS) and electrophoretic light scattering (ELS)

Z-average mean hydrodynamic diameter (d_H_) and polydispersity index (PdI) were measured by Dynamic Light Scattering (Malvern, Zetasizer Nano 90 S) (4 mV He-Ne ion laser, λ = 633 nm, scattering angle: 90°). The sample was prepared from a TNG stock solution in water (10 mg/mL, prepared with sonication at 40% amplitude for 30 s) by dilution with 1 mM KCl (1.0 mg/mL). In addition, the ζ potential of TNG was measured by Electrophoretic Light Scattering (Malvern, Zetasizer Nano ZC).

#### Cryogenic transmission electron microscopy (cryo-TEM)

Cryo-TEM analysis was carried out using a Tecnai F20 X TWIN microscope (FEI Company, Hillsboro, Oregon, USA). Images were recorded with a Gatan Rio 16 CMOS 4k camera (Gatan Inc., Pleasanton, California, USA) and processed with Gatan Microscopy Suite (GMS) software (Gatan Inc., Pleasanton, California, USA). Specimens were prepared from TNG dispersion in DI water (500 µg/mL) via the vitrification of aqueous solutions on oxygen plasma-activated grids with holey carbon film (Quantifoil R 2/2; Quantifoil Micro Tools GmbH, Großlöbichau, Germany).

#### Nuclear magnetic spectroscopy (NMR)

^1^H NMR spectra were recorded in deuterated solvents by using Varian NMR instrument operating at 600 MHz. Chemical shifts are reported in ppm (δ) relative to tetramethylsilane (DMSO_d6_) or 3-(trimethylsilyl)propionic-2,2,3,3-d_4_ acid sodium salt (D_2_O) as an internal reference.

#### Determination of conjugated trehalose (CTre)

The content of trehalose in nanogels was determined after the alkaline hydrolysis of ester bonds in trehalose acrylate units. Briefly, 40 µL of 1 M NaOH was added to 400 µL of nanogel dispersion (100 µg/mL) in PBS (pH 7.4) and the mixture was incubated at 70 ºC for 1 h. After neutralization with 40 µL of 1 M HCl, the sample was subjected to enzymatic determination of trehalose by using Trehalose Assay Kit in a microplate assay procedure. CTre (% w/w) was calculated as the percentage of weight of trehalose in nanogel vs. weight of nanogel.

#### Trehalose release study by enzymatic determination

An initial stock dispersion of nanogels (10 mg/mL) was prepared by redispersing nanogel powder in DI water followed by ultrasonication (40% amplitude, 30 s). The stock was diluted to a final concentration of 100 µg/mL (30 mL) in PBS (pH 7.4) containing 1% v/v antibiotic antimycotic solution in a 50-mL glass vial. Afterward, the nanogel dispersion was placed in an incubator at 37 °C with constant shaking (332 ×*g*). Aliquots (800 µL) were taken every 24 h over 30 days and frozen at -20 ºC. After all samples were collected, they were thawed and the amount of trehalose was determined enzymatically.

#### Colloidal stability of nanogels in various media

Colloidal stability of nanogels (1.0 mg/mL, containing 1% v/v antibiotic antimycotic solution) was determined for 7 days at 37 °C in various biological media, including DI water, PBS (pH 7.4), NS, DMEM, and DMEM + 10% FBS, by measuring the optical density (OD) at 650 nm on a microplate reader. The OD value was converted to % transmittance using the following equation:

$$\% \, {\text{Transmittance}}={\text{antilog}} (2 - {\text{OD}})$$

#### Hemolytic rate of trehalose-releasing nanogels

Fresh blood was collected from the auricular vein of healthy New Zealand white rabbits. The blood was then diluted with saline at a ratio of 4:5. In this experiment, three types of samples were prepared: a negative control (normal saline), a positive control (ultrapure water), and test samples (TNG at different concentrations). Each sample (1 mL) was immersed in a water bath at 37 °C for 30 min. After that, 20 µL of the diluted rabbit blood was added to each tube.

The tubes were further incubated in a water bath at 37 °C for 1 h. Following the incubation, the samples were centrifuged at 500 ×*g* for 5 min. The supernatant was carefully removed, and photographs were taken. The collected supernatant was then used to measure OD at a wavelength of 545 nm. The OD value was converted to % hemolytic rate using the following equation:

$$\% {\text{Hemolytic rate}} = \frac{{OD}_{t}-{OD}_{nc}}{{OD}_{pc}-{OD}_{nc}}\times 100\text{\%}$$

Where *OD*_*t*_ is the OD test value obtained in the presence of trehalose or TNG, *OD*_*nc*_ is the negative control and the *OD*_*pc*_ is the positive control.

#### In vitro cytotoxicity of trehalose-releasing nanogels

The cell harmlessness of TNG is a prerequisite for biological research. The MTS Cell Proliferation and Cytotoxicity Detection Kit was used to measure cytotoxicity of TNG according to the manufacturer’s instructions. Briefly, HUVECs were inoculated at a density of 1 × 10^4^ per well in 96-well plates. After 24 h, various concentrations of TNG (25, 100, 150, 200 µg/mL) were added, and the cells were co-cultured for 24 h. Then, 10 µL of MTS was added to each well and incubated for additional 2–4 h, and finally OD value at 450 nm was measured. The OD value was converted to % cell viability using the following equation:

$$\% {\text{Cell viability}} =\frac{{OD}_{t}-{OD}_{b}}{{OD}_{c}-{OD}_{b}}\times 100\text{\%}$$

Where *OD*_*t*_ is the OD test value obtained in the presence of TNG, *OD*_*c*_ is the OD test value obtained in the absence of TNG, and the *OD*_*b*_ is the OD test value of blank plate.

#### In vitro cellular uptake of trehalose-releasing nanogels

RAW264.7 cells were cultured on 20-mm round coverslip until cell adherence to ~ 50%. Then the medium was removed and replaced with fresh serum-free culture medium containing free trehalose and TNG labeled with Cy5 which have the equal trehalose concentration. After 1 or 3 h of co-incubation, the medium was removed, washed with PBS for 3 times, then fixed with 4% paraformaldehyde for 15 min, washed with PBS for 3 times again, the nuclei were finally labeled with DAPI and observed under confocal microscope.

#### In vitro autophagy stimulation

Raw 264.7 cells were cultured on 20-mm round coverslip at a density of 1 × 10^5^ and in 6-well plates until cell adherence to ~ 50%. Then the medium was removed and replaced with fresh serum-free culture medium containing 1 µg/mL of lipopolysaccharide (LPS) and 40 µg/mL of oxidized low-density lipoprotein (ox-LDL) for 12 h to induce the formation of foam cells.

Trehalose (100 µM) or TNG (at a concentration equivalent to 100 µM of free trehalose) was added to the above foam cells, incubated for 12 h, then washed 3 times with PBS at 4 °C. Cells in 96-well plates were lysed with RIPA lysis buffer and protease inhibitor (phenylmethylsulfonyl fluoride, PMSF) were added and placed at 4 °C. After manual scrapping, the lysates were collected and centrifuged at 15,000 ×*g* for 20 min. Then, the loading buffer was added to the supernatants at a 1:4 ratio, mixed, and then boiled for 5 min before being stored at -80 °C. The sequestosome 1 (p62) and microtubule-associated protein 1 A/1B-light chain 3 (LC3) protein expressions were quantified by Western blot.

Additionally, cells on the round coverslip were also washed three times with PBS at 4 °C, fixed with 4% paraformaldehyde for clarity, and then incubated overnight at 4 °C with LC3 rabbit polyclonal antibody or p62/SQSTM1 rabbit polyclonal antibody. After rinsing the round coverslips five times with PBST (0.1% Tween-20 PBS), they were incubated for 1 h with fluorescently-labeled rabbit secondary antibodies under darkness. Subsequently, the coverslips were washed five times before the nuclei were finally labeled with DAPI. Fluorescence signals from DAPI, p62, and LC3 were detected by SP8 confocal microscopy.

#### Intracellular ROS clearance in macrophages

RAW264.7 cells were cultured in 12-well plates for 12 h. After cells were pretreated with trehalose (100 µM) or TNG (at a concentration equivalent to 100 µM of free trehalose) for 4 h, they were stimulated with 0.5 µM H_2_O_2_ for 0.5 h. The negative control group was treated with fresh medium, and the positive control group was only stimulated with 0.5 µM H_2_O_2_ for 0.5 h. Subsequently, cells were rinsed and treated with DCFH-DA (10 µM) in serum-free DMEM for 30 min. After washing with PBS and the intracellular ROS clearance was observed in cell culture dishes by fluorescence microscope. Through similar procedures, the cells were harvested in PBS, intracellular fluorescent signals were measured via flow cytometry (CytoFLEX, Beckman Coulter) and analyzed using FlowJo software.

#### In vitro lipid efflux

Raw 264.7 cells were inoculated in 6-well plates at a cell density of 2 × 10^5^ per well. Cells were then stimulated with 1 µg/mL of LPS and 40 µg/mL of ox-LDL to form foam cells. The cells were treated with free trehalose (100 µM) or TNG (at a concentration equivalent to 100 µM of free trehalose) for 24 h. Following this, the medium was removed, and the cells were washed twice with PBS before being fixed using a 4.0% paraformaldehyde (PFA) solution. Subsequently, the cells were incubated with Oil Red O (ORO) isopropanol working solution for 15 min. Then cells were observed by optical microscopy. In addition, the intracellular ORO was extracted by isopropanol and the ORO concentration was determined by measuring its absorbance at 524 nm via an UV-Vis spectroscopy.

#### Animal models

Male apolipoprotein E-deficient (ApoE^−/−^) mice, aged eight weeks, were obtained from the Hunan SJA Bioscience Co., Ltd. (Hunan, China). All animal care and experimental protocols comply with the relevant laws, regulations and standards concerning animal welfare ethics. This project has been supervised and approved by Laboratory Animal Welfare and Ethics Committee of Chongqing University (IACUC issue number: COU-IACUC-RE-202109-002).

#### Atherosclerosis treatment with nanogels

ApoE^−/−^ mice were randomized into 3 groups (5 mice/group), and high fat diet (HFD) was given for 12 weeks. Then, the mice were subjected to different treatments for one month. The mice were injected with 0.9% saline as the untreated control group, while the other two groups were treated with either free trehalose at a dose of 2.5 g/kg of trehalose, or TNG at a concentration of 16 mg/kg every three days via tail vein injection. The body weight of mice was monitored during the treatment.

#### In vivo pharmacokinetics evaluation of nanogels

To evaluate the in vivo pharmacokinetics of TNG, the Cy5-labeled TNG (Cy5-TNG) was intravenously administered to C57BL/6 mice at dose of 16 mg/kg, while free Cy5 was intravenously injected at the equal concentration of Cy5 in Cy5-TNG. Then, 20 µL of blood was collected at 0.5, 1, 2, 4, 8, 12, and 24 h after injection. The blood samples were diluted with 40 µL PBS contained EDTA2K in 96-well black plates, and the fluorescence intensity was measured by fluorescence microplate reader (Hitachi, Japan).

#### In vivo accumulation of nanogels in atherosclerotic lesion and biodistribution

After atherosclerosis modeling, mice were injected with 150 µL Cy5 (control group) or Cy5-TNG via tail vein. After 24 h, the mice were euthanized, and the aortas were isolated after perfusion with 0.9% saline containing heparin sodium. Additionally, heart, liver, spleen, lung, and kidney were harvested to analyze the biodistribution of nanogels in the main organs. Imaging and fluorescence quantification were performed using the Xenogen IVIS 200 system.

#### Efficacy study and histological study of atherosclerotic plaques after treatment

Quantitative analysis of atherosclerotic plaques after treatments: After the 10th round of trehalose and TNG treatments via intravenous injections (IV), the aortas from ApoE^−/−^ mice were harvested, spanning from the heart to the iliac bifurcation. Aortas were fixed by perfusion with 4% paraformaldehyde, dissected longitudinally, and then stained with Oil Red O (ORO) to quantify the plaque area. The extent of atherosclerotic plaque at the aortic root was also determined by the same way. Quantitative analysis of atherosclerotic plaque areas was performed using Photoshop 2020 software.

The aortic roots were fixed with 4% paraformaldehyde in PBS for 1 h. Frozen sections were prepared from the fixed samples, and ORO staining was performed to quantify the plaque area. For immunofluorescence analysis, the sections were washed with PBS and then permeabilized/blocked using a solution containing 0.5% Triton-X100 in 5% BSA. Antibodies specific to p62, LC3 and CD68 were separately incubated with the sections overnight at 4 °C. After washing the sections five times with PBST (PBS with 0.1% Tween-20), they were incubated with secondary antibodies for 1 h. Nuclei were stained with DAPI in the dark. The fluorescence signals from DAPI, p62, LC3 and CD68 were detected using SP8 confocal microscopy (Leica, Germany). Sections of the main organs including heart, liver, spleen, lung, and kidney were analyzed by hematoxylin-eosin (HE) staining.

After one month of treatments, a complete blood routine analysis and serum biochemistry analysis were conducted. Blood samples were collected and analyzed using an automated hematology analyzer (Sysmex KX-21, Sysmex Co., Japan) to obtain the complete blood count (CBC) data. The concentrations of various biochemical markers, including alanine aminotransferase (ALT), aspartate aminotransferase (AST), creatinine (CR), uric acid (UA), high-density lipoprotein (HDL), low-density lipoprotein (LDL), triglyceride (TG), and total cholesterol (TC) in the serum, were quantified using an automated analyzer platform (Roche Cobas C501, Roche Co., Switzerland).

### Statistical analysis

The collected data were presented as mean ± *SD* (*n ≥* 3). The statistical analysis was performed using GraphPad Prism Version 8.4.3 software (GraphPad, USA). Tukey’s test and one-way analysis of variance (ANOVA) were employed to identify group differences. To determine whether there is a significant difference between two specific groups, an unpaired *t*-test (two tails) was used. The significance thresholds for differences were set at *p < 0.05, **p < 0.01, ***p < 0.001, ****p < 0.0001, and *ns*, no significance.

### Electronic supplementary material

Below is the link to the electronic supplementary material.

Supplementary Material 1

## Data Availability

All data contained in the study are in this article.
