# Correction: Nanogels with covalently bound and releasable trehalose for autophagy stimulation in atherosclerosis

**DOI:** 10.1186/s12951-023-02290-7

**Published:** 2024-01-17

**Authors:** Yuan Zhong, Ali Maruf, Kai Qu, Małgorzata Milewska, Ilona Wandzik, Nianlian Mou, Yu Cao, Wei Wu

**Affiliations:** 1 Key Laboratory for Biorheological Science and Technology of Ministry of Education, State and Local Joint Engineering Laboratory for Vascular Implants, Bioengineering College, Faculty of Medicine, Chongqing University, Chongqing, 400030 China; 2 Department of Organic Chemistry, Bioorganic Chemistry and Biotechnology, Faculty of Chemistry, Silesian University of Technology, Krzywoustego 4, Gliwice, 44-100 Poland; 3 Biotechnology Center, Silesian University of Technology, Krzywoustego 8, Gliwice, 44-100 Poland

**Correction to:** ***Journal of Nanobiotechnology*** **(2023) 21:472**

10.1186/s12951-023-02248-9

In this article the statement in the Funding information section was incorrectly given as ‘National Natural Science Foundation of China (31,971,301, 32,171,324),’ and should have read ‘National Natural Science Foundation of China (31971301, 32171324)’.

The original article [1] has been corrected.
