# Supplementary material for: Nanogels with covalently bound and releasable trehalose for autophagy stimulation in atherosclerosis
Source: J Nanobiotechnology. 2023 Dec 8;21:472. doi: 10.1186/s12951-023-02248-9 (PMC10704736; doi:10.1186/s12951-023-02248-9)
Supplement: Supplementary file 1 — Supplementary Material 1 [file 12951_2023_2248_MOESM1_ESM.docx]

***Supplementary Information***

*for*

**Nanogels with covalently bound and releasable trehalose for autophagy stimulation in atherosclerosis**

Yuan Zhong ^1,†^, Ali Maruf ^2,3,†^, Kai Qu ^1^, Małgorzata Milewska ^2,3^, Ilona Wandzik ^2,3,*^, Nianlian Mou ^1^, Yu Cao ^1^, Wei Wu ^1,*^

^1^ Key Laboratory for Biorheological Science and Technology of Ministry of Education, State and Local Joint Engineering Laboratory for Vascular Implants, Bioengineering College, Faculty of Medicine, Chongqing University, Chongqing 400030, China

^2^ Department of Organic Chemistry, Bioorganic Chemistry and Biotechnology, Faculty of Chemistry, Silesian University of Technology, Krzywoustego 4, 44-100 Gliwice, Poland

^3^ Biotechnology Center, Silesian University of Technology, Krzywoustego 8, 44-100 Gliwice, Poland

^†^ The authors contribute equally to this work

* Corresponding authors. E-mail addresses: [david2015@cqu.edu.cn](mailto:david2015@cqu.edu.cn) (WW), ilona.wandzik@polsl.pl (IW)

**Formulation of nanogels**

**Table S1.** Formulation of nanogels based on moles and mass feeding.

| Samples | Formulation based on moles and mass  (mmol (mg)) | | | | | |
| --- | --- | --- | --- | --- | --- | --- |
|  | TreA | HEA | AM | 4-AMBA | 4-AMBA-sulfo-NHS | MBA |
| **TNG** | 0.385 (152.7) | - | 0.496 (35.3) | 0.085 (13.4) | - | 0.130 (20.0) |
| **HEA_1_NG** | - | 0.385 (44.8) | 0.496 (35.3) | 0.085 (13.4) | - | 0.130 (20.0) |
| **HEA_2_NG** | - | 1.315 (152.7) | 0.496 (35.3) | 0.085 (13.4) | - | 0.130 (20.0) |
| **NHS-TNG** | 0.385 (152.7) | - | 0.496 (35.3) | 0.085 (13.4) | 0.011 (4.0) | 0.130 (20.0) |

TNG: trehalose-releasing nanogels, HEA_1_NG and HEA_2_NG: 2-hydroxyethyl acrylate-containing nanogels, HEA: 2-hydroxyethyl acrylate, AM: acrylamide, 4-AMBA: 4-acrylamidobutanoic acid, TreA: 6-O-acryloyl-α,α’-trehalose, MBA: *N,N'*-methylenebisacrylamide.

**Colloidal stability of TNG, HEA_1_NG and HEA_2_NG in different biological media for 7 days**

**Figure S1.** Colloidal stability of TNG, HEA_1_NG and HEA_2_NG in different biological media for 7 days**.** TNG: trehalose-releasing nanogels, HEA_1_NG and HEA_2_NG: 2-hydroxyethyl acrylate-containing nanogels.

**Synthesis of 4-acrylamidobutanoic acid 3-sulfo-*N*-hydroxysuccinimide ester sodium salt (4-AMBA-Sulfo-NHS)**


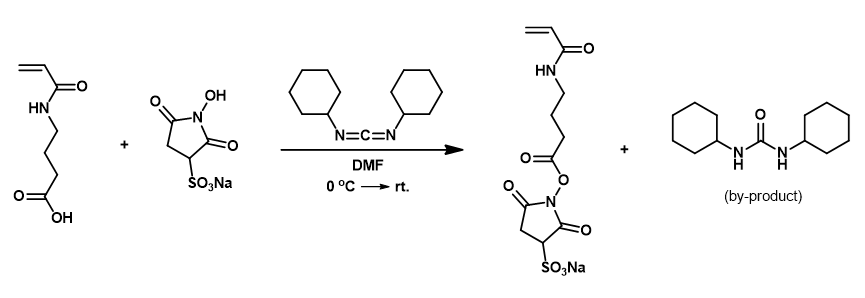


4-AMBA-Sulfo-NHS was synthesized based on the method reported by Tsuji et al. (2019) for the synthesis of homologous *N*-sulfosuccinyl-6-hexyloylacrylamide sodium salt. Briefly, sulfo-NHS (434.3 mg, 2.0 mmol) and 4-AMBA (314.3 mg, 2.0 mmol) were placed in a 25 mL round-bottom flask and dissolved in anhydrous DMF (6 mL) under argon atmosphere while stirring. The flask was then cooled in an ice bath for 15 min. A DCC solution (833.6 mg, 4.0 mmol) in 2.0 mL of anhydrous DMF was added dropwise into the mixture and kept 1 h in an ice bath, followed by another 20 h at room temperature. On the next day, the reaction mixture was transferred to the refrigerator (4 ºC) for 30 min followed by filtration through Celite and washing with 3 mL of DMF. The product was then precipitated with 140 mL of diethyl ether, centrifuged at 14610 ×g (4 ºC, 2 min) and washed four times with 20 mL of diethyl ether. The white precipitate was then dried under reduced pressure to give the final product (4-AMBA-Sulfo-NHS) with 49% yield.

**Reference:** S. Tsuji, Y. Aso, H. Ohara, and T. Tanaka, “Polymeric water-soluble activated esters: synthesis of polymer backbones with pendant N-hydoxysulfosuccinimide esters for post-polymerization modification in water,” Polym J, vol. 51, no. 10, pp. 1015–1022, Oct. 2019, doi:10.1038/s41428-019-0221-4.

**^1^H NMR** (DMSO-*d*_6_, 600 MHz) δ [ppm]: 8.18 (t, *J* = 5.6 Hz, 1H, –N**H**C(O)–); 6.20 (dd, *J* = 17.1, 10.2 Hz, 1H, –C**H**=CH_2_); 6.08 (dd, *J* = 17.1, 2.2 Hz, 1H, –CH=C**H**_2_ *trans*); 5.58 (dd, *J* = 10.2, 2.2 Hz, 1H, –CH=C**H**_2_ *cis*); 3.95 (bs, 1H, >C**H**SO_3_Na); 3.24–3.08 (m, 3H, –NHC**H_2_**–, –C**H_a_**H_b_CHSO_3_Na); 2.86 (dd, *J* = 18.4, 2.4 Hz, 1H, –CH_a_**H_b_**CHSO_3_Na); 2.75–2.65 (m, 2H, –C**H_2_**C(O)O–); 1.83–1.73 (m, 2H, –CH_2_C**H_2_**CH_2_–). **^13^C NMR** (DMSO-*d*_6_, 150 MHz) δ [ppm]: 168.77, 165.33 (2x –**C**(O)N<, –**C**(O)O–); 164.67 (–NH**C**(O)–); 131.68 (–CH=**C**H_2_); 125.03 (–**C**H=CH_2_); 56.27 (>**C**HSO_3_Na); 37.57 (–NH**C**H_2_–); 30.91 (–**C**H_2_CHSO_3_Na); 27.86 (–**C**H**_2_**C(O)O–); 24.26 (–CH_2_**C**H**_2_**CH_2_–).

**^
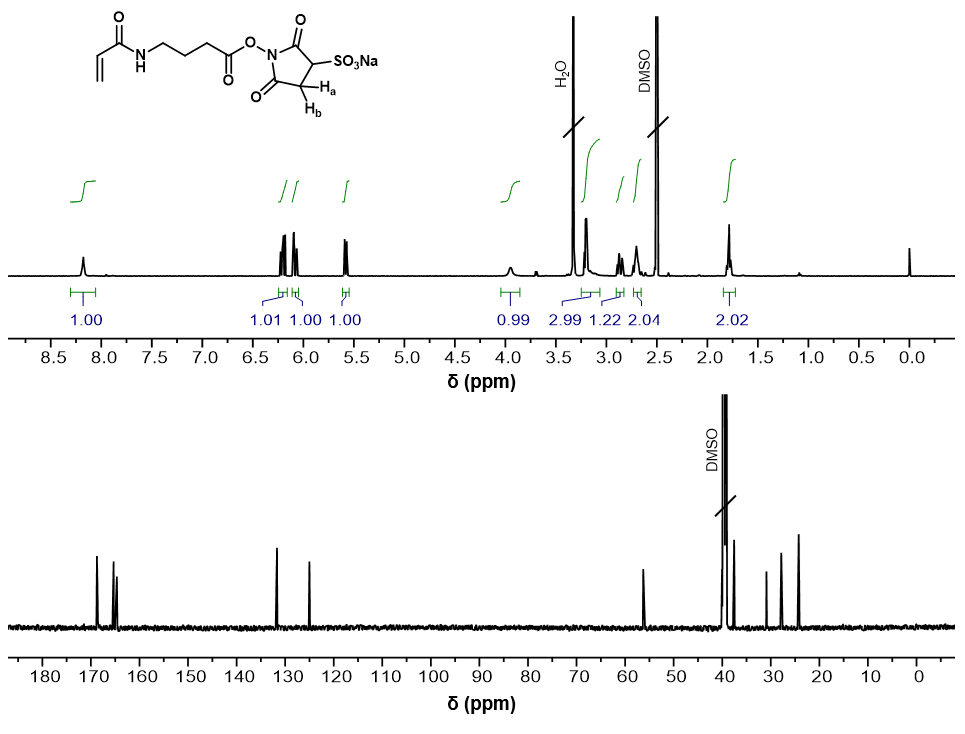
^**

**Figure S2.** ^1^H (600 MHz) and ^13^C NMR (150 MHz) spectra of 4-acrylamidobutanoic acid 3-sulfo-*N*-hydroxysuccinimide ester sodium salt.

**Actual conjugated trehalose**

**Figure S3.** Actual conjugated trehalose compared to the recipe. TreA feeding and free trehalose feeding were determined based on the recipe. Actual conjugated trehalose was determined by enzymatic assay. Data are presented as mean ± *SD* (*n* = 4).

**Hemolysis profile of TNG**


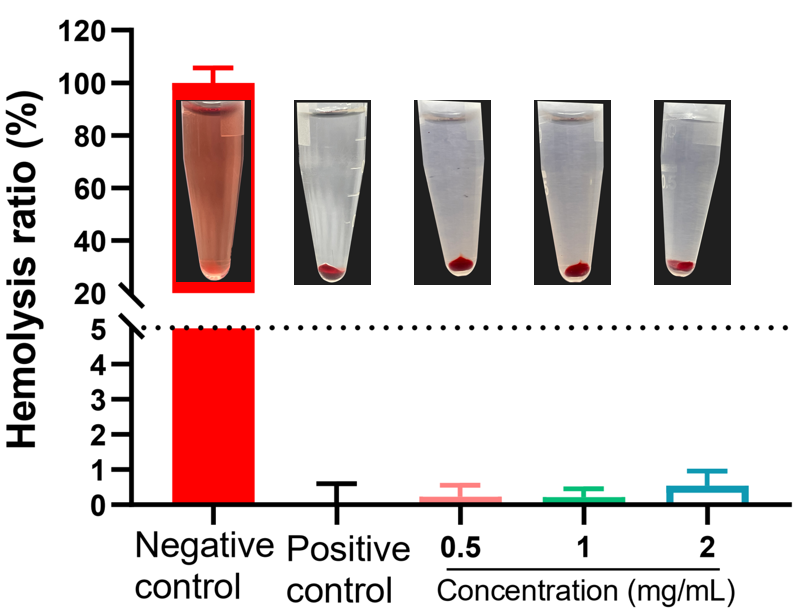


**Figure S4.** Hemolysis profile of trehalose-releasing nanogels (TNG) at different concentrations (0.5, 1.0, and 2.0 mg/mL) in red blood cells. Data are presented as mean ± *SD* (*n* = 3).

**Cytotoxicity profile of TNG**


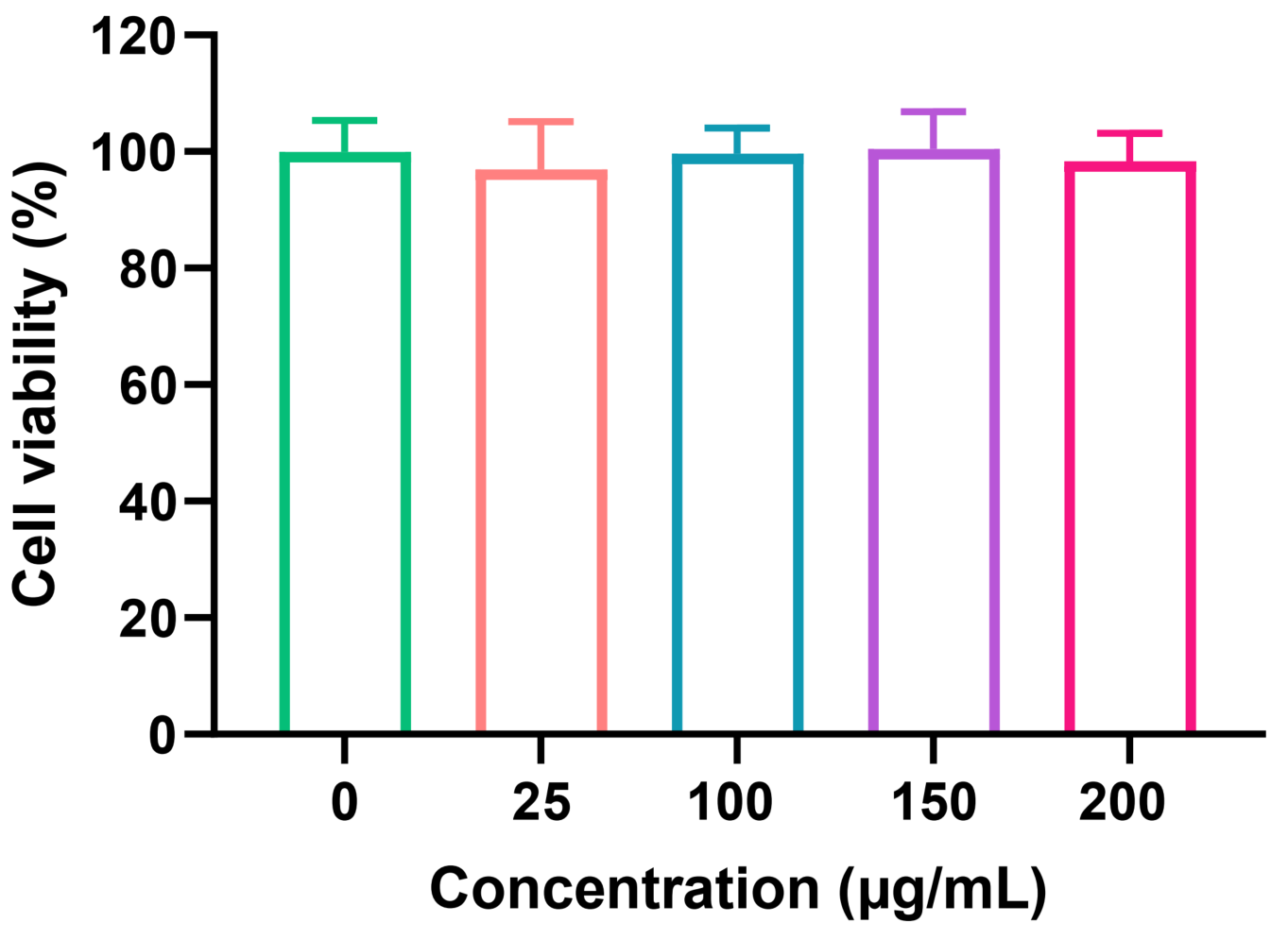


**Figure S5.** HUVECs viability after treatments with trehalose-releasing nanogels (TNG) at different concentrations (0, 25, 100, 150, and 200 µg/mL) for 24 h. Data are presented as mean ± *SD* (*n* = 5).

**Cellular uptake of TNG**

**
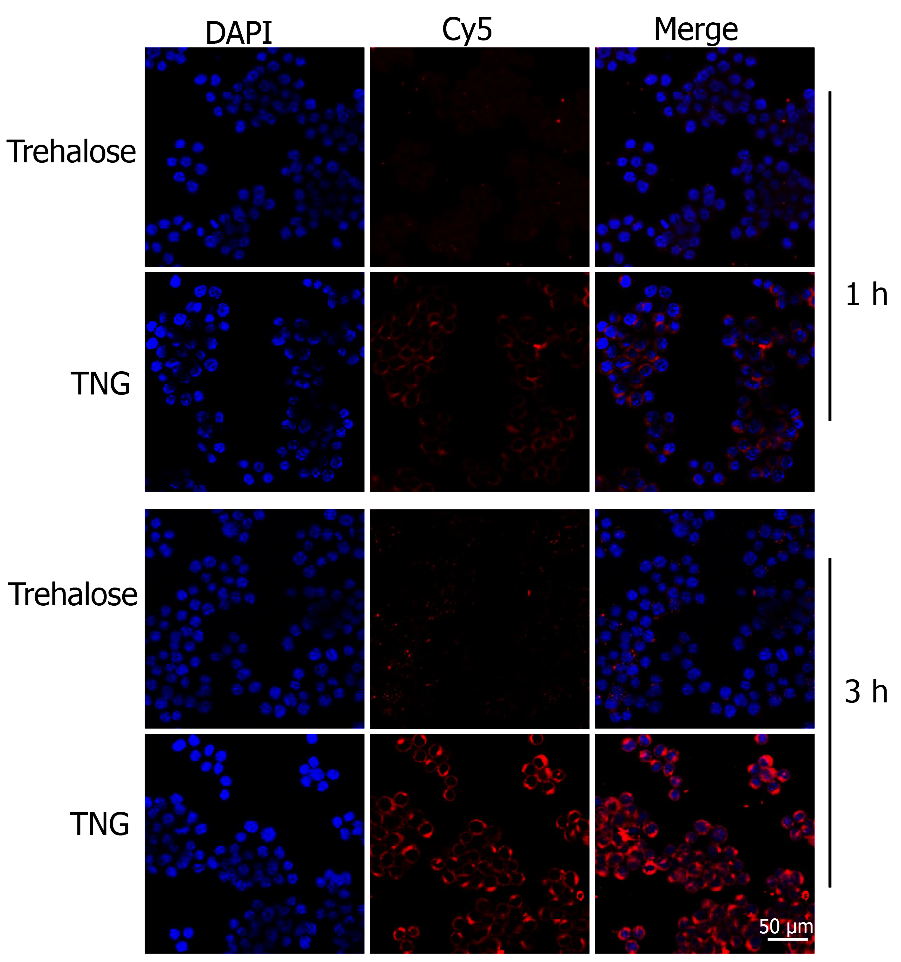
**

**Figure S6.** Confocal microscopy images of time-dependent cellular uptake of Cy5-TNG or trehalose. After RAW264.7 cells were incubated with Cy5-TNG or trehalose for various periods of time, nuclei were stained with DAPI (blue), Cy5 was red, scale bar: 50 µm.

***In vivo* pharmacokinetics evaluation of nanogels in mice**

**Figure S7.** *In vivo* pharmacokinetics evaluation of nanogels in mice. TNG was fluorescently conjugated with Cy5. Cy5-TNG was intravenously injected at concentration of 16 mg/kg, while free Cy5 was injected at the equal concentration of Cy5 in Cy5-TNG.

**CD68 expression in atherosclerosis plaque after one-month treatment**

**
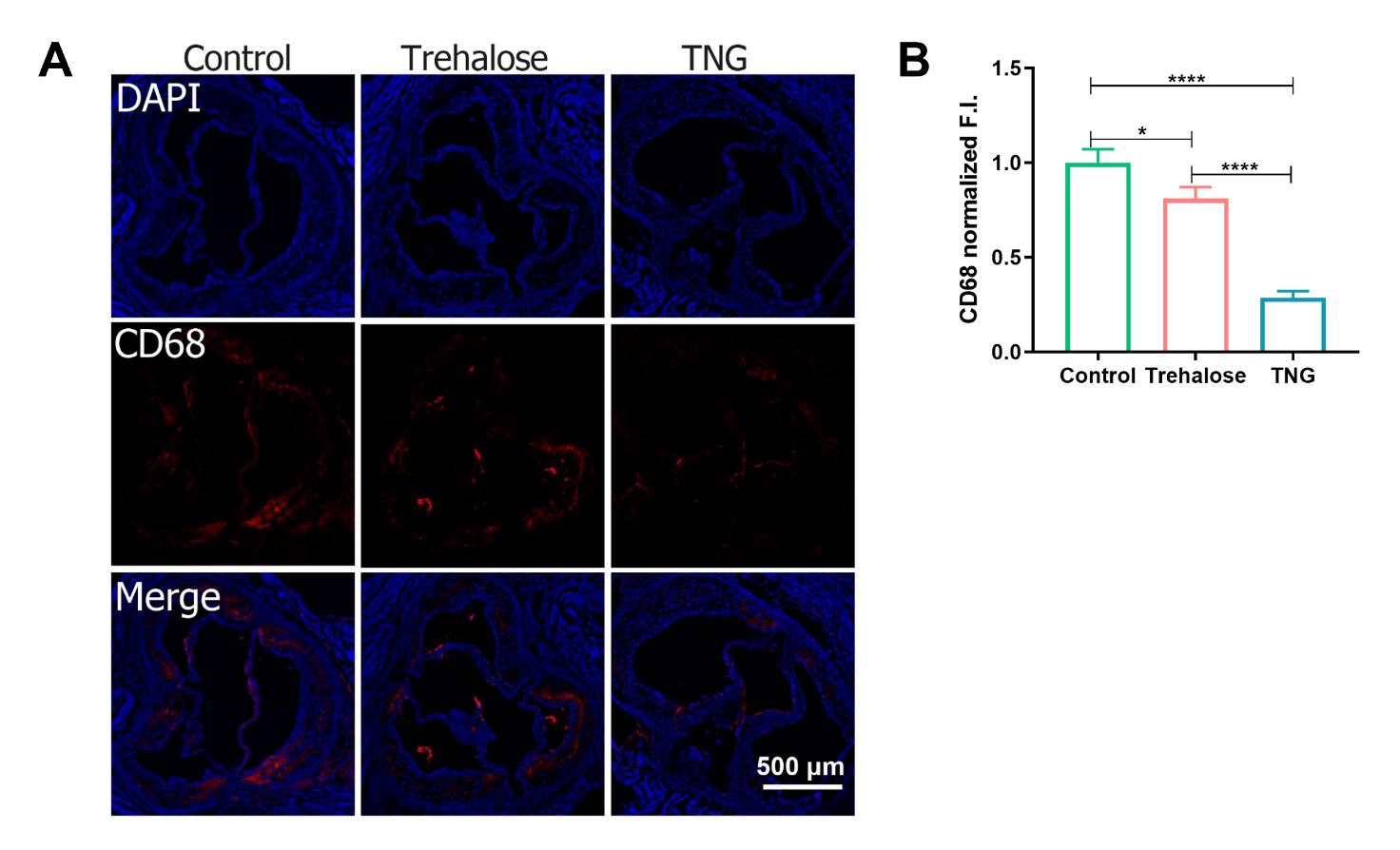
**

**Figure S8.** (A) CLSM images and (B) quantification of CD68 (red fluorescence) expressions in atherosclerotic plaques of the aortic root sections in ApoE^−/−^ mice after one month of treatments with free trehalose and TNG compared to the control, scale bars: 500 µm.

**In vivo biosafety assessment**

**
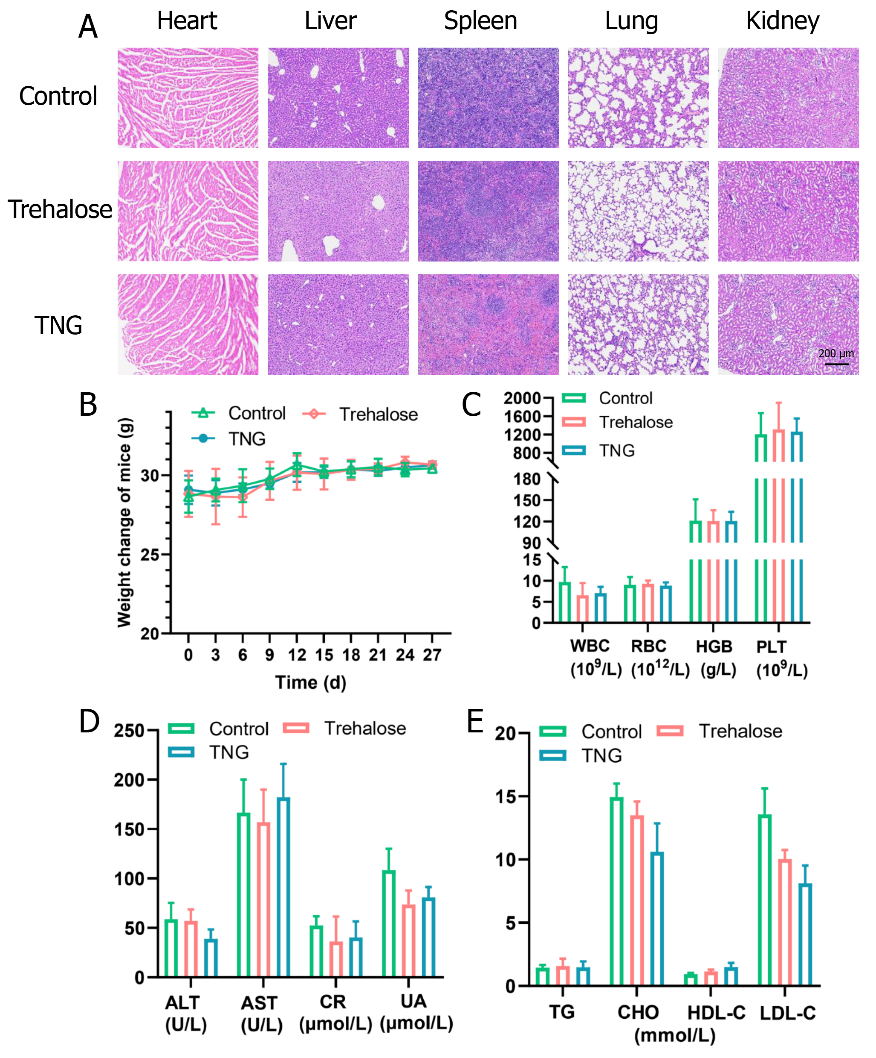
**

**Figure S9.** *In vivo* biosafety assessment of trehalose-releasing nanogels. (**A**) H&E-stained images of main organs from ApoE^−/−^ mice after various treatments for one month. All the micrographs were acquired at 100× magnification, scale bar: 200 µm. (**B**) The body weight of ApoE^−/−^ mice monitored over one month of treatments. (**C**) Complete blood count (CBC) profile of ApoE^−/−^ mice after one month of treatments. WBC: white blood cells, RBC: red blood cells, HGB: hemoglobin, and PLT: platelets. (**D**) The biochemical assays of hepatic and kidney functions of ApoE^−/−^ mice after one month of treatments. ALT: alanine aminotransferase, AST: aspartate aminotransferase, CR: creatinine, and UA: uric acid. (**E**) Blood lipid profile of ApoE^−/−^ mice after one month of treatments. TG: triglycerides, CHO: total cholesterols, HDL-C: high-density lipoprotein (HDL) cholesterols, LDL-C: low-density lipoprotein (LDL) cholesterols. Data are presented as mean ± *SD*, *n* = 5.

Table S2. Comprehensive blood routine analysis and normal reference ranges in mice.

| Samples | WBC (10^9^/L) | RBC (10^12/^L) | HGB (g/L) | PLT (10^9^//L) |
| --- | --- | --- | --- | --- |
| Reference range | 0.8-10.6 | 6.5-11.5 | 110-165 | 400-1600 |
| Control | 9.70±3.49 | 9.02±1.85 | 121.00±30.21 | 1207.60±461.50 |
| Trehalose | 6.54±2.88 | 9.22±0.84 | 120.60±15.42 | 1308.80±587.78 |
| TNG | 7.02±1.53 | 8.80±0.78 | 120.40±13.13 | 1260.80±290.49 |

Table S3. Serum liver and kidney function indicators detection data and normal reference ranges in mice.

| Samples | ALT (U/L) | AST (U/L) | CR (μmol/L) | UA (μmol//L) |
| --- | --- | --- | --- | --- |
| Reference range | 10.06-96.47 | 36.31-235.48 | 10.91-84.09 | 44.42-224.77 |
| Control | 58.95±16.28 | 166.51±33.61 | 52.37±9.35 | 108.52±21.54 |
| Trehalose | 57.09±11.56 | 156.98±33.01 | 36.23±25.32 | 73.69±14.09 |
| TNG | 38.99±9.48 | 182.23±33.60 | 40.33±16.23 | 80.93±10.42 |

Table S4. Serum lipid indicators detection data and normal reference ranges in mice.

| Samples | TG (mmol/L) | CHO (mmol/L) | HDL-C (mmol/L) | LDL-C (mmol//L) |
| --- | --- | --- | --- | --- |
| Reference range | 0.84-2.72 | 2.05-4.16 | 1.28-2.65 | 0.12-0.26 |
| Control | 1.43±0.22 | 14.94±1.06 | 0.94±0.09 | 13.58±2.05 |
| Trehalose | 1.57±0.59 | 13.49±1.10 | 1.14±0.15 | 10.04±0.72 |
| TNG | 1.47±0.47 | 10.62±2.24 | 1.49±0.33 | 8.13±1.39 |
